# Inter-individual relationships within a Canadian SPOR research network: a social network study

**DOI:** 10.1186/s12913-022-08343-1

**Published:** 2022-07-27

**Authors:** Justin Lawarée, James M. Bowen, Joyce Dogba, Valeria E. Rac, Mathieu Ouimet

**Affiliations:** 1 École nationale d’administration publique, Quebec, Canada; 2 Diabetes Action Canada, Toronto, Canada; 3 Program for Health System and Technology Evaluation, Toronto, Canada; 4 Université Laval, Quebec, Canada

**Keywords:** Research networks, SPOR networks, Patient engagement, Translational research, Social network analysis

## Abstract

**Background:**

Efforts have been made by health research granting agencies to bring research closer to patients’ concerns. In Canada, such efforts were formalized in 2011 with the funding of the Strategy for Patient-Oriented Research (SPOR)’s research networks to address research priorities identified by patients and accelerate the translation of research findings into patient care and health care policy. Among these networks, SPOR Diabetes Action Canada (DAC) has created patient-partner circles to facilitate their integration within the network. The nature of the relationships within this atypical patient-oriented research network is systematically explored in this paper.

**Methods:**

A cross-sectional social network study was conducted among the SPOR DAC’s network members to examine inter-individual interactions, and the topics discussed the most between members. Descriptive data analyses were conducted to explore which discussion topics were discussed most among members whose primary roles were research, administration, governance, and patient representation.

**Results:**

The response rate was 51.9%, providing data on 76.5% of the maximum number of connections in the network. The survey captured 2763 inter-individual relationships. Responses to a sub-question inserted in the survey show that 482 of these relationships (17,4%) existed before joining the network in collaboration on a research project. Most ties captured in the survey were yearly or quarterly, while few relationships were monthly, weekly, or daily. In measured relationships, members discussed several topics, the most frequent being scientific research, patient engagement, network coordination and governance, and operations and management. The topics associated with the most significant proportion of relationships captured in the survey were scientific research (45.4%) and patient engagement (40.7%). Management & operations and governance & coordination follow, corresponding to 24.3 and 23.9% of the captured relationships. All discussion topic subnetworks were either somewhat or highly centralized, meaning that relationships were not equally distributed among members involved in these discussions. Of the 1256 relationships involving exchanges about scientific research, 647 (51.5%) involved a researcher, 419 (33.3%) an administrator, 182 (14.5%) a patient partner, and 82 (6.5%) a member whose primary role is network governance.

**Conclusions:**

Scientific research and patient engagement were the most common topics discussed, consistent with the patient-centered research at the heart of the SPOR Diabetes Action Canada network. The study identified several relationships where a patient partner has discussed scientific research with a researcher. However, relationships involving research discussions were three times more common between a researcher and an administrator than between a researcher and a patient partner, although twice as many patient partners as administrators participated in the survey. The institutionalization of patient-partner involvement in large research networks is an evolving practice for which optimal engagement methods are still being explored.

**Supplementary Information:**

The online version contains supplementary material available at 10.1186/s12913-022-08343-1.

## Background

One of the critical challenges of health research is the “valley of death,” referring to the chasm between basic and applied research [1]. The importance of bridging this gap has been enshrined in official strategies of national granting agencies in several countries, including the United States [2] and Canada [3]. This issue is not new [4] and paved the way for translational research [5–8] and implementation science [9] to promote improved evidence-informed clinical, management, and policy-making practices. The involvement of health services consumers [10–14] and policymakers [15] in setting research priorities and in the very process of knowledge production [11, 13, 16–18] are among the many proposed solutions to the translational problem.

Translational health research networks [19–22] bring together several stakeholders beyond scientists who are funded to find solutions to complex problems that often span beyond the healthcare system. They aim to foster the development of health technologies (diagnostic tests, therapies, devices, procedures, care pathways, etc.) and facilitate the implementation of research findings into policy and practice to improve the life of patients and their families. These research networks are interdisciplinary, multifunctional, and funded by governmental and non-governmental partners. Many of their members are not researchers (i.e., patients and informal caregivers, administrators, clinicians, decision-makers, etc.). The extraordinary complexity of these membership-based research networks poses the challenge of promoting and coordinating the relationships between network members with various backgrounds and sometimes different personal and professional interests. Few studies described the structure and content of the relationships within research networks with such atypical features. Understanding the functioning of these networks necessitates examining the inter-individual relationships that make them up. It has been done for a translational cancer research network in Australia [20, 21, 23, 24], but to our knowledge, not yet for a Canadian Strategy for Patient-Oriented Research (SPOR) research network.

Health research in Canada is primarily funded by an organization with an explicit mission to encourage translational research. Indeed, the legal assignment of the Canadian Institutes of Health Research (CIHR) is “to excel, according to internationally accepted standards of scientific excellence, in the creation of new knowledge *and its translation* [emphasized by us] into improved health for Canadians, more effective health services and products and a strengthened Canadian health care system.” [25] In 2011, a little more than 10 years after its inception, CIHR launched Canada’s Strategy for Patient-Oriented Research which consists in a “collaboration of researchers, patients, provinces and territories, health care professionals and others – all working in partnership to integrate research into patient care, ultimately improving the health of Canadians.” [10, 26, 27] Patients’ involvement in all aspects of the research is among the core principles of SPOR, along with a 1:1 funding matching formula with non-federal government partners. Among the core elements of SPOR, the SPOR *research networks* focus on specific health areas deemed to be a priority in multiple provinces and territories [27]. The SPOR networks address research priorities identified by patients and accelerate the translation of research findings into patient care and health care policy. Within each network, the intent is that patient partners are engaged and contribute to defining network priorities and the research process at the project level. Funded over 5 years with the possibility of renewal, seven SPOR networks were launched in 2016 involving more than 130 partners and totaling $240 million (Canadian dollars) in funding [26].

An understanding of how the CIHR SPOR initiative has supported patient engagement within the new research networks is of importance moving forward. This topic of discussion should theoretically be the subject of a fair number of interactions within such networks, along with scientific research. To the best of our knowledge, no study has examined, within a SPOR network, the topics of discussion between different types of members such as researchers, patient partners, network administrators, and those whose primary role is network governance. This study fills this gap.

The study provides the first systematic description of relationships within a SPOR research network using some of the techniques that were used to analyze a translational cancer research network [20, 21, 23, 24]. More precisely, this paper presents findings of two consecutive cross-sectional surveys that were part of a larger network evaluation project for SPOR Diabetes Action Canada (SPOR DAC), one of the five Canadian chronic disease SPOR research networks. The main objective of the study was to measure the relative frequency with which topics related to the mission of SPOR networks were discussed among SPOR DAC members with different primary roles (i.e., researchers, patient partners, administrators, and individuals in charge of network governance) and from different locations at the time of the second survey. Another objective of the study was to describe the general characteristics of the relationships based on data of both surveys. Few empirical studies have measured the topics of discussion in translational research networks.

## Method

### Setting

SPOR DAC was launched in 2016, and was awarded $12,500,000 (Canadian dollars) in funding from the Canadian Institutes of Health Research (CIHR). In addition to the CIHR funding, matching funding has been obtained, equaling $22,406,479, from various organizations, corporations, and private donors until March 31, 2022.

The research network’s scientific programming includes several projects whose ultimate goal is to prevent and reduce diabetes-related complications among people with diabetes and their families. SPOR DAC’s quadruple aim goals are to improve patient experience, population outcome, health professional experience, and health system cost “with a vision to transform the health trajectory for all Canadian men, women, and children with diabetes at risk for complications*”* [28]. The research network comprises patient partners, clinical and non-clinical researchers, research coordinators, and administrators from multiple Canadian provinces, but mainly from the two most populous ones, Ontario and Quebec. In 2019, listed network members were affiliated with at least 33 organizations, including 21 Canadian universities, four federal or provincial government agencies, five non-governmental organizations, and three companies.

Like other SPOR networks, SPOR DAC has adopted a network administrative organization (NAO) governance model. This governance model is centralized around an administrative entity that acts as a facilitator and a broker. The DAC SPOR network is administered by an executive director, administrative team, and Operation/Management Committee. Also, it has a steering council that monitors the work of the different network components. The NAO governance model is considered appropriate for networks of moderate to large size, with medium density, and in which consensus on objectives is moderately high [29].

Canada ranks fourth after Russia, China, and the United States in land area, spanning a distance of 5514 km, with 10 provinces and 3 territories, each responsible for their healthcare systems. When conducting the surveys, the SPOR-DAC research network had members from the Atlantic provinces (e.g., New Brunswick) to the western provinces (Alberta and British Columbia). However, almost three-fourths of the network members were from Ontario and Quebec, the two most populous provinces with 38.4 and 23.0% of the population in 2021, respectively [30]. The administrative hub of the network is located in Toronto (Ontario), while formal patient engagement activities were mainly coordinated from Quebec City (Quebec). Moreover, it should be noted that the two principal investigators on the grant application were based in these two provinces.

SPOR DAC’s research activities are coordinated through goal-oriented groups and enabling programs. At the network’s launch, the network’s activities were organized into eight goal groups. Since its inception, the network has evolved and grown by creating new groups/programs. The research network currently includes six research goal groups (i.e., Retinopathy Screening, Indigenous People’s Health, Innovations in Type 1 Diabetes, Digital Health for Diabetes Research and Care, Foot-care to Prevent Amputations, and Aging Community and Population Health) and five enabling programs (i.e., Patient Engagement, Training and Mentoring, Knowledge Translation, Sex and Gender, and Health Technology Assessment and Network Analytics). Patient partners, who form an integral part of the network, are organized into three advisory circles: the Collective Patient Circle, the Indigenous Patients Circle, and the Francophone and New Immigrants Circle.

### Design and units of analysis

The study design is a cross-sectional population-based social network study covering up to the network’s third and fourth year of operation. The survey was conducted in two successive years, with the first covering from April 1st, 2018 to March 31, 2019 and the second examining relationships from April 1st, 2019 to March 31, 2020. Initially, it was planned to conduct three successive surveys to capture the evolution of the linkage structure of the network. Unfortunately, we could not perform the third survey due to financial constraints and the pandemic.

As sampling units, official members refer to all individual stakeholders (patient partners, researchers, administrative employees, chair council members, co-leads, etc.) who were on the administrative list of network members by March 2019 (first survey) and March 2020 (second survey). They received a link to the web survey platform (Qualtrics) that allows filling the questionnaire on a computer, a tablet, or a smartphone. We designed the cross-sectional surveys as a whole-network study measuring the links between all pairs of individuals in the network. In the first survey, we conducted four recalls. In contrast, in the second survey, due partly to the Covid-19 pandemic, we did six recalls and gave participants additional time to complete the questionnaire.

Defining network boundaries is a critical step in social network analyses [31–34]. We used the exhaustive (and updated) list of official members of SPOR DAC since the aim was to measure relationships within 1 year and not at a particular event (e.g., during the annual workshop). More precisely, the positional approach used to define network boundaries was the fixed-list technique based on the administrative dataset updated up to March 31, 2019 (first survey) and up on March 31, 2020 (second survey). The lists of members were provided to us by the DAC’s administrative team. They included 150 individuals for the first survey and 185 for the second survey. We invited only those on these lists to participate in the surveys.

### Survey instrument and measurement

Participants responded to sociometric questions designed to measure inter-individual relationships in the network (see the Supplementary file). To facilitate memorization, we placed the picture of each member next to their name and reminded the temporal length of the last fiscal year beside each name. The first question aimed to measure the perceived frequency of the relationship, while the second question measured the content of the discussions between each pair of individuals. In the second survey, we added a sub-question to the frequency question to determine the proportion of relationships that existed before joining the network, a strategy employed in studying an Australian translational cancer research network [21]. Before answering the sociometric questions, participants were provided with the definition of interaction used in the study: any recalled direct conversation between two individuals, of any length, through any means of communication (e.g., face-to-face, telephone, email, chatting, texting), that occurred within the context of DAC SPOR-related activities between April 1, 2018, and March 31, 2019 (first survey), and between April 1, 2019, and March 31, 2020 (second survey).

We measured the frequency of the interactions on the six-point frequency scale as follows: (i) daily, multiple times a day, most days in the last 12 months; (ii) weekly, multiple times a week, most weeks in the last 12 months; (iii) monthly, from time to time, most months in the last 12 months; (iv) quarterly, a few times during the last 12 months; or (v) yearly, only once during the last 12 months, (vi) no contact during the last 12 months. For each interaction, respondents were also asked if, before joining the network, they had previously collaborated with the person on a research project (e.g., clinical trials, funded projects, quality improvement initiatives).

A sociometric question dealt with the discussion topics with each contact identified through the frequency question. We asked participants to indicate which of eight topics they had discussed during their conversations with that person (see Table 1). We adapted the list of topics from a published social network analysis of health care networks [35–37] and the Quadruple Aim goals of SPOR DAC. An “other” box allowed respondents to answer the following question for logistic reasons even if they did not thick any listed topics.

**Table 1:** Topics of discussion measured in surveys

Discussion Topic	Definition	Examples
Scientific research	It refers to the research process or traditional ways to disseminate research findings.	Grant’s application, protocol writing, data collection, data analysis, or publications, conferences.
Training and mentoring	It refers to student supervision, training, or any other educational activity.	Mentoring, teaching, or participation in professional training.
Patient Engagement	It refers to patient partners’ (PP)’ participation within the DAC SPOR Network.	Participation of PPs in research committees, collaboration in the research process, management, or recruitment of PPs.
Management and operations	It refers to the management of projects or activity implementation.	Recruitment, reporting, coordination of services and resources.
Governance and coordination	It refers to the strategic decision-making for the whole Network or specific groups.	Defining the mission and objectives of the DAC SPOR Network, program evaluation, and planning, creating a new group, monitoring projects, or coordinating researchers.
Commercialization of research findings	It refers to the commercialization of research products.	Licensing, consultancy, intellectual property protection, or technology transfer processes.
Transfer of research findings	It refers to disseminating research results to external actors, excluding the commercialization of research and scientific publications.	Guidelines, policy briefs, media articles, or presentations to decision-makers.

### Data treatment and analysis

The data were analyzed using *Gelphi*. Network data were considered symmetric or undirected, meaning that when a given respondent indicated that he or she had a relationship with a listed member while that listed member did not confirm the relationship either because he or she did not participate in the survey or because he or she could not confirm the connection for other reasons (e.g., could not recall), the relationship was entered in the database. We symmetrized the relational frequency data by selecting the highest frequency score between each pair of members (Schoen et al., 2014). For discussion topics, all relationships were also transformed into undirected ties.

For the purposes of describing the frequency of the relationships, we have focused for this on the results from both surveys. For reasons related to participation rates and the fact that the two data points represent two successive years, the discussion topics analysis was based on the most recent survey data after 4 years of the network creation. Descriptive statistics were computed for the frequency of relationships captured in the survey. We calculated the number and percentage of members with at least one captured exchange for each discussion topic. We calculated the number and percentage of links between members of different provinces or regions and between members of the same province or region for each discussion topic. We also calculated the number and percentage of links between members of different groups (researchers & patient partners, researchers & administrators, etc.) and between members of the same group. This allowed us to observe to what extent patient partners who are supposed to be critical players in a SPOR network were engaged in the various facets of the network’s life, such as discussions about scientific research, training and mentoring, management and operations, governance, and research transfer.

We computed the centralization of each discussion topic subnetwork based on the degree of centrality of each member (i.e., their number of direct relationships). Each subnetwork includes only members with at least one inter-individual discussion about the topic. The centralization degree is a normalized measure that takes a value between 0 and 1, where 1 indicates that relationships are concentrated around a single actor, while 0 means that the number of connections is equally distributed among the members of the subnetwork.

### Ethics

Ethics approval was obtained from the Université Laval’s Research Ethics Board (*2018–336-A-1/08-05-2019*) and the Research Ethics Board at the University Health Network (19–5773).

## Results

### Sample characteristics, participation rate, and overall network features

In 2019, the official membership of the network based on administrative data included 150 individuals, of which there were 99 researchers (66%), 26 patient partners (17.3%), 11 (7.3%) administrators and 11 (7.3%) members whose primary role was network governance. In 2020, the official membership of the network has increased to 185 individuals, of which there were 126 researchers (68.1%), 36 patient partners (19.5%), 15 administrators (8.1%), and 8 (4.3%) members whose primary role was network governance.

In this study, the units of analysis are the inter-individual relationships, not the participants or the network’s members. In undirected social network analysis, the participation rate that applies to relationships is always higher than the one for the respondents [31]. In a study where the direction of the relationship does not matter, it only takes one of the two individuals forming a relationship to capture that relationship. At the respondent level, the response rate (see Table 2) increased by 9.2 percentage points from the first (42.7%) to the second (51.9%) survey. The second survey thus provides data on 76.5% of the maximum number of connections in the network. Participation rate has increased significantly in all categories of membership except for administrators. It is notable that the participation rate for patient partners increased by 16 percentage points from the first to the second survey. Data from the 2020 survey show that the highest participation rates were among administrators (73.3%), those whose primary role was governance (62.5%), and patient partners (58.3%). Researchers were the group that participated least in the survey (46.8%). Given that they represented 68.1% of the network’s members, their lower participation rate lowered the overall participation rate.

**Table 2:** Participation rate among types of functions (2019 & 2020)

	Distribution size (2019)	Distribution size (2020)	Completed (2019)	Completed (2020)	Participation Rate (2019)	Participation Rate (2020)
Administration	11	15	10	11	90.9%	73.3%
Governance	11	8	6	5	42.8%	62.5%
Research	99	126	37	59	37.4%	46.8%
Patient Partner	26	36	11	21	42.3%	58.3%
Total	150	185	64	96	42.7%	51.9%

Administrative and survey data did not identify the location of 25 (13.6%) of the 185 network members, that were mostly patient partners for which we did not have their province of residence. The 2020 data indicate that slightly more than half of the members were located in Ontario (*n* = 96; 51.9%), 40 (21.6%) in Quebec, 12 (6.5%) in Alberta or British Columbia, 6 (3.2%) in Manitoba or Saskatchewan, and the same number in the Maritime provinces.

**Fig. 1:**
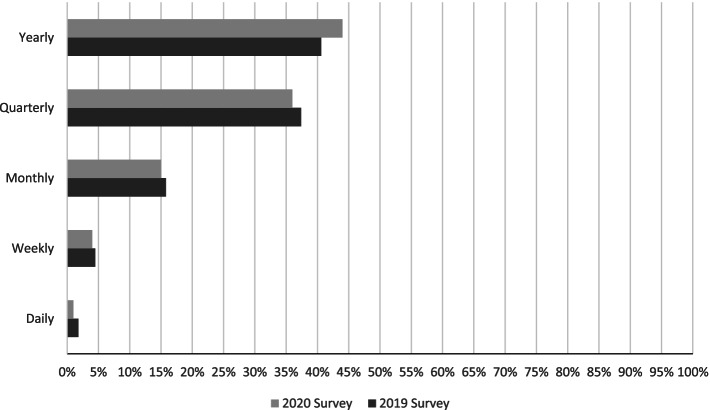
Distribution of inter-individual relationships based on the relational frequency in 2019 and 2020

The network’s density up to 2020 was 0.16 (0.20 in 2019), which means that at least 16% of the possible relationships were captured and activated. Members’ average number of relationships within the network was 29.9 ties (30.7 in 2019). None of the 150 (2019 survey) and 185 (2020 survey) members were isolated, meaning that all members were part of at least one relationship captured in the survey. In the 2019 survey, 2305 inter-individual relationships were captured, while the 2020 survey captured 2763 inter-individual relationships. Responses to a sub-question inserted in the 2020 survey (see the Supplementary file) show that 482 of these relationships (17,4%) existed before joining the network in the form of a collaboration on a research project (e.g., clinical trials, funded projects, quality improvement initiatives). Regarding the frequency of captured relationships, most ties captured in the surveys were yearly or quarterly, while few relations were on a monthly, weekly, or daily basis (see Fig. 1).

### Discussion topics within the network

The topic associated with the most significant proportion of the network relationships captured in the 2020 survey was scientific research (45.4%). Of the 185 network members, 174 (94%) were involved in at least one relationship to discuss this topic (see Table 3). Patient engagement is not far behind, as 40.7% of the relationships captured in the survey involved exchanges about this topic, and 87% of network members were involved in such discussions.

**Table 3:** Frequency statistics for discussion topics

Topic	N (%) of captured relationships that involved exchanges about the topic	Number/total (%) of network members involved in at least one relationship for the topic
Scientific research	1256/2763(45.4%)	174/185 (94%)
Patient engagement	1126/2763 (40.7%)	161/185 (87%)
Training and mentoring	499/2763 (18.1%)	145/185 (78.4%)
Governance and coordination	660/2763 (23.9%)	163/185 (88.1%)
Management and operations	673/2763 (24.3%)	163/185 (88.1%)
Commercialization of research	33/2763 (1.2%)	32/185 (17.3%)
Transfer of research findings	446/2763 (16.1%)	143/185 (77.3%)

Management & operations and governance & coordination follow, corresponding to 24.3 and 23.9% of the captured relationships. Relationships involving exchanges on either of these two topics involved 163 of the 185 members (88.1%). Training & mentoring and the transfer of research findings to non-academic audiences were discussed in 18.1 and 16.1% of the relationships captured, respectively. Relationships that included exchanges on training & mentoring and the transfer of research findings involved 145 (78.4%) and 143 members (77.3%), respectively. As for the commercialization of research, it was the least discussed topic in the relationships captured in the survey. This topic was discussed in only 33 of the 2763 measured relationships (1.2%). From the 185 members, only 32 (17.3%) were involved in a relationship where commercialization was discussed.

All the discussion topic subnetworks are either somewhat or highly centralized, meaning that relationships were far from equally distributed among members involved in these discussions. The most heavily centralized subnetworks were those concerning discussions about the commercialization of research (0.87), governance & coordination (0.73), and management & operations (0.72). Discussion subnetworks about scientific research (0.44), patent engagement (0.42), training & mentoring (0.47), and transfer of research findings to non-academic audiences (0.42) were nonetheless somewhat centralized.

### Distribution of discussion topics according to the member’s primary role

One of the particularities of a SPOR network is that its membership is not limited to researchers but also includes patient partners, administrators, and members involved in the governance of the network. It thus becomes relevant to examine the proportion of relationships that involve each type of member for each topic of discussion and the proportion of relationships between members with different roles. Table 4 reports these data.

**Table 4:** Within and between-group distribution of discussion topics

	Topics						
Dyads	Scientific research	Patient engagement	Training and mentoring	Management and operations	Governance and coordination	Commercialization	Transfer of research findings
	N (%)	N (%)	N (%)	N (%)	N (%)	N (%)	N (%)
	Relations between members of different primary role						
R < --> PP	111 (8.8)	364 (32.3)	61 (12.2)	33 (5.0)	33 (4.9)	1 (3.0)	43 (9.6)
R < --> A	312 (24.8)	124 (11.0)	127 (25.5)	302 (45.8)	137 (20.4)	12 (36.4)	95 (21.3)
R < --> G	64 (5.1)	45 (4.0)	18 (3.6)	23 (3.5)	149 (22.1)	3 (9.1)	42 (9.4)
PP < --> A	27 (2.1)	139 (12.3)	28 (5.6)	44 (6.7)	33 (4.9)	0 (0.0)	3 (0.7)
PP < --> G	4 (0.3)	45 (4.0)	5 (1.0)	4 (0.6)	42 (6.2)	0 (0.0)	3 (0.7)
A < --> G	11 (0.9)	8 (0.7)	4 (1.0)	12 (1.8)	45 (6.7)	0 (0.0)	3 (0.7)
	Relations between members of the same primary role						
R < --> R	647 (51.5)	201 (17.9)	214 (42.9)	172 (26.1)	174 (25.9)	11 (33.3)	225 (50.4)
PP < --> PP	40 (3.2)	173 (15.4)	21 (4.2)	8 (1.2)	11 (1.6)	0 (0.0)	9 (2.0)
A < --> A	37 (2.9)	22 (2.0)	19 (3.8)	51 (7.7)	27 (4.0)	6 (18.2)	18 (4.0)
G < --> G	3 (0.2)	5 (0.4)	2 (0.4)	11 (1.7)	22 (3.3)	0 (0.0)	5 (1.1)
**Total**	**1256 (100)**	**1126 (100)**	**499 (100)**	**660 (100)**	**673 (100)**	**33 (100)**	**446 (100)**
Involving R	1134 (90.3)	734 (65.2)	420 (84.2)	530 (80.3)	493 (73.2)	27 (81.8)	405 (90.8)
Involving PP	182 (14.5)	721 (64.0)	115 (23.0)	89 (13.5)	119 (17.7)	1 (3.0)	58 (13.0)
Involving A	419 (33.3)	293 (26.0)	178 (35.7)	409 (62.0)	242 (35.9)	18 (54.5)	119 (26.7)
Involving G	82 (6.5)	63 (5.6)	29 (5.8)	50 (7.6)	258 (38.3)	0 (0.0)	53 (11.9)

Note: *R* Researcher; *PP* Patient partner; *A* Administrator; *G* Member whom primary role is governance

Of the 1256 relationships involving exchanges about scientific research, 647 (51.5%) involved a researcher, 419 (33.3%) an administrator, 182 (14.5%) a patient partner, and 82 (6.5%) a member whose primary role is network governance. Over half of the relationships involving discussions about scientific research were between two researchers (51.5%), while a quarter of these exchanges took place between a researcher and an administrator (24.8%). Scientific research was discussed between a researcher and a patient partner in 8.8% of the 1256 relationships involving exchanges on this topic.

Almost the same proportion of the 1126 relationships where patient engagement was discussed involved a researcher (65.2%) or a patient partner (64%), while just over a quarter of these relationships (26%) involved an administrator. As with scientific research, few of the relationships where patient engagement was discussed involved a member providing governance to the network (5.6%). Of the 1126 relationships involving discussions about patient engagement, 364 (32.3%) were between a researcher and a patient partner, while 201 (17.9%) and 173 (15.4%) of such relationships were between two researchers and two patient partners, respectively. On the other hand, 12.3% of the relationships with discussions about patient engagement were between an administrator and a patient partner, while 11% involved an administrator and a researcher.

Regarding training and mentoring, 420 of the 499 relationships with discussions about this topic (84.2%) involved a researcher, 178 (35.7%) an administrator, 115 (23%) a patient partner, and 29 (5.8%) a person responsible for network governance. Of these 499 relationships, 214 (42.9%) were between two researchers, while a quarter (127; 25.5%) were between a researcher and an administrator. Training and mentoring were discussed between a researcher and a patient partner in 61 relationships (12.2%).

Discussions about management and operations involved mainly researchers and administrators. More precisely, 530 of the 660 relationships (80.3%) in which this topic was discussed involved a researcher, and 409 (62%) an administrator. A patient partner and a member in charge of network governance were involved in 89 (13.5%) and 50 (7.6%) of such exchanges. This topic was discussed between a researcher and an administrator in 302 of 660 relationships involving this topic (45.8%). Moreover, 172 (26.1%) of the relationships where management and operations were discussed were between two researchers.

Researchers were heavily engaged in discussion about network governance and coordination, as shown by the fact that 493 of the 773 relationships (73.2%) involving this topic of discussion were between two researchers. Although the number of actors whose primary role is to provide network governance is small (8 out of 185), 258 (38.3%) of the 673 relationships about this discussion topic involved one of these individuals. This proportion was 35.9% (*n* = 242) for exchanges involving an administrator. A quarter of the relationships about governance and coordination were between two researchers (*n* = 174; 25.9%), while one fifth was between a researcher and an administrator (*n* = 137; 20.4%) and between a researcher and a person in charge of network governance (*n* = 149; 22.1%).

The large majority of the 33 captured inter-individual ties related to the commercialization of research involved a researcher (*n* = 27; 81.8%), over half involved a network administrator (*n* = 18; 54.5%), and over one third were between a researcher and an administrator (*n* = 12; 36.4%). One-third of these relationships were between two researchers (*n* = 11; 33.3%), while 18.2% (*n* = 6) were between two network administrators. As for the transfer of research results not involving commercialization, the inter-individual discussions were dominated by researchers. From the 446 relationships comprising discussions about this topic, 405 (90.8%) involved a researcher, while 225 (50.4%) were between two researchers. A little more than one-fourth of these relationships involved a network administrator (*n* = 119; 26.7%), and one-fifth were between a researcher and an administrator (*n* = 95; 21.3%).

### Geographical distribution of discussion topics

The data presented in Table 5 indicates that Ontario is not just the administrative hub of the network. For all of the discussion topics examined in the survey, more than 50% of the relationships involved at least one member from Ontario. Quebec was also an important focal point for inter-individual exchanges within the network. For every topic of discussion outside of the management & operations and the commercialization of research, half or more of the relationships involved a member located in Quebec, although members from Quebec represented one-fifth of the survey participants.

**Table 5:** Geographical distribution of discussion topics

	Topics						
Dyads	Scientific research	Patient engagement	Training and mentoring	Management and operations	Governance and coordination	Commercialization	Transfer of research findings
	N (%)	N (%)	N (%)	N (%)	N (%)	N (%)	N (%)
	Relations between members of different provinces or regions						
O < --> Q	234 (18.6)	220 (19.5)	88 (17.6)	110 (16.7)	194 (28.8)	9 (27.3)	107 (24.0)
O < --> ABC	97 (7.7)	25 (2.2)	13 (2.6)	56 (8.5)	41 (6.1)	1 (3.0)	20 (4.5)
O < --> M	9 (0.7)	17 (1.5)	39 (7.8)	21 (3.2)	23 (3.4)	0 (0.0)	9 (2.0)
O < --> MS	37 (2.9)	25 (2.2)	10 (2.0)	21 (3.2)	12 (1.8)	2 (6.1)	6 (1.3)
O < --> U	34 (2.7)	137 (12.2)	9 (1.8)	27 (4.1)	32 (4.8)	0 (0.0)	7 (1.6)
Q < --> ABC	29 (2.3)	30 (2.7)	4 (0.8)	13 (2.0)	24 (3.6)	1 (3.0)	9 (2.0)
Q < --> M	9 (0.7)	31 (2.8)	38 (7.6)	12 (1.8)	21 (3.1)	0 (0.0)	6 (1.3)
Q < --> MS	16 (1.3)	25 (2.2)	11 (2.2)	6 (0.9)	15 (2.2)	1 (3.0)	4 (0.9)
Q < --> U	58 (4.6)	197 (17.5)	29 (5.8)	14 (2.1)	35 (5.2)	0 (0.0)	16 (3.6)
ABC < --> M	3 (0.2)	4 (0.4)	6 (1.2)	1 (0.2)	1 (0.1)	0 (0.0)	1 (0.2)
ABC < --> MS	5 (0.4)	4 (0.4)	2 (0.4)	4 (0.6)	2 (0.3)	0 (0.0)	0 (0.0)
ABC < --> U	3 (0.2)	11 (1.0)	0 (0.0)	4 (0.6)	4 (0.6)	0 (0.0)	0 (0.0)
M < --> MS	2 (0.2)	2 (0.2)	4 (0.8)	1 (0.2)	3 (0.4)	0 (0.0)	0 (0.0)
M < --> U	2 (0.2)	19 (1.7)	12 (2.4)	1 (0.2)	7 (1.0)	0 (0.0)	2 (0.4)
MS < --> U	3 (0.2)	11 (1.0)	1 (0.2)	1 (0.2)	2 (0.3)	0 (0.0)	0 (0.0)
	Relations between members of the same province or region						
O < --> O	568 (45.2)	151 (13.4)	126 (25.3)	308 (46.7)	152 (22.6)	17 (51.5)	177 (39.7)
Q < --> Q	110 (8.8)	152 (13.5)	84 (16.8)	49 (7.4)	90 (13.4)	0 (0.0)	77 (17.3)
ABC < --> ABC	15 (1.2)	5 (0.4)	6 (1.2)	6 (0.9)	5 (0.7)	2 (6.1)	4 (0.9)
M < --> M	3 (0.2)	3 (0.3)	7 (1.4)	2 (0.3)	2 (0.3)	0 (0.0)	1 (0.2)
MS < --> MS	1 (0.1)	3 (0.3)	2 (0.4)	0 (0.0)	0 (0.0)	0 (0.0)	0 (0.0)
U < --> U	18 (1.4)	54 (4.8)	8 (1.6)	3 (0.5)	8 (1.2)	0 (0.0)	0 (0.0)
**Total**	**1256 (100)**	**1126 (100)**	**499 (100)**	**660 (100)**	**673 (100)**	**33 (100)**	**446 (100)**
Involving O	979 (77.9)	575 (51.1)	285 (57.1)	543 (82.3)	454 (67.4)	29 (87.9)	326 (73.1)
Involving Q	744 (59.2)	655 (58.2)	254 (50.9)	204 (30.9)	379 (56.3)	11 (33.3)	219 (49.1)
Involving ABC	152 (12.1)	79 (7.0)	31 (6.2)	74 (11.2)	77 (11.4)	4 (12.1)	34 (7.6)
Involving M	28 (2.2)	76 (6.7)	106 (21.2)	38 (5.7)	57 (8.5)	0 (0.0)	19 (4.3)
Involving MS	64 (5.1)	70 (6.2)	30 (6.0)	33 (5.0)	34 (5.0)	3 (9.1)	10 (2.2)
Involving U	118 (9.4)	429 (38.1)	59 (11.8)	50 (7.6)	88 (13.1)	0 (0.0)	25 (5.6)

Note: *O* Ontario; *Q* Quebec; *ABC* Alberta & British Columbia; *M* Maritimes; *MS* Manitoba & Saskatchewan; *U* Unknown location

Of the 1256 relationships involving exchanges about scientific research, 979 (77.9%) involved a member from Ontario and 744 (59.2%) a member from Quebec. Forty-five percent (*n* = 568) of the relationships involving discussion about scientific research were between two members from Ontario, while 18.6% (*n* = 234) of these exchanges were between a member from Ontario and one from Quebec. Most of the inter-individual relationships about scientific research captured in the survey involved members of these two provinces (see Table 5), knowing that these two provinces represent about three-quarters of the network’s membership.

Of the 1126 relationships where patient engagement was discussed, 655 (58.2%) involved a member from Quebec and 575 (51.1%) from Ontario. It should be noted that 429 (38.1%) of the relationships about this topic involved a member from an unknown location (i.e., survey and administrative data for location were missing for 25 members). About one-fifth of the captured relationships (*n* = 220; 19.5%) for discussion about patient engagement were between a member from Quebec and one from Ontario.

Compared to Ontario and Quebec, few members were from the Maritimes, but the hub for the network’s training program was located in the Maritime Provinces. Relationships involving discussion about training and mentoring involved mostly members from Ontario (*n* = 285; 57.1%), Quebec (*n* = 254; 50.9%) and the Maritimes (*n* = 106; 21.2%). In 126 (25.3%) of the 499 relationships involving discussions about training and mentoring, the exchanges were between two members of Ontario, while 84 (16.8%) of these relationships were between two members from Quebec. Exchanges about training and mentoring were held between a member from Ontario and one from Quebec in 88 (17.6%) of the relationships involving this discussion topic.

Activities related to the management and operations were centrally coordinated from Toronto. As can be seen at the bottom of Table 5, of the 660 relationships involving discussion about this topic, 543 (82.3%) involved a member from Ontario. Moreover, nearly half of the captured relationships related to this topic were between two members from Ontario (*n* = 308; 46.7%). As for the 673 relationships comprising discussions about governance and coordination, 454 (67.4%) and 379 (56.3%) involved a member from Ontario and Quebec, respectively. Of these 673 relationships, over a quarter (*n* = 194; 28.8%) were between a member of Ontario and Quebec, while 152 (22.6%) were between two members of Ontario.

The few relationships about the commercialization of research have primarily involved members in Ontario and Quebec. Indeed, 29 of these 33 relationships (87.9%) involved a member from Ontario and one-third (*n* = 11; 33.3%) from Quebec. Half of these relationships were between two Ontario members, and 27.3% (*n* = 9) were between a member in Ontario and one in Quebec. As for the transfer of research results not involving commercialization, the inter-individual discussions were also dominated by Ontario and Quebec members. From the 446 relationships comprising discussions about this topic, 326 (73.1%) involved a member from Ontario and 219 (49.1%) a member from Quebec. Of these relations, 39.7% (*n* = 177) were between two Ontario members, 24% (*n* = 107) between a member from Ontario and one located in Quebec, and 17.53% (*n* = 77) between two members from Quebec.

## Discussion

This study was the first to describe the content of the relationships in a SPOR research network. Specifically, the study’s main objective was to examine the relative frequency with which topics related to the mission of SPOR networks were discussed among SPOR DAC members with different primary roles and from different locations at the time of the second survey. Our cross-sectional data showed that all discussion topic subnetworks were somewhat or highly centralized around highly connected members. Further evaluation is needed to determine if the uneven distribution of relationships in the discussion subnetworks is observed in other similar networks. The study found that scientific research and patient engagement were the most common topics discussed, consistent with the patient-centered research at the heart of the SPOR Diabetes Action Canada network. Although fewer in number, the relationships in which two members discussed management & operations or governance & coordination were nevertheless numerous. While they involved few patient partners, several researchers have been involved in these exchanges with other researchers or administrators. The importance of network management and coordination is one of the features of large networks governed under the network administrative organization (NAO) governance model [29].

Most of the relationships captured were quarterly or annual, and very few occurred monthly, weekly, or daily. While this could be seen as a weakness of the network, it could also simply reflect that many researchers are engaged in multiple other research programs and other types of activities such as teaching. In addition, monthly, weekly, and daily relationships occur mainly with colleagues working in the same unit, while network members come from various Canadian organizations and locations. Moreover, this study showed that in terms of membership and relationships between members, the focal point of this pan-Canadian research network was Ontario and, to a lesser extent Quebec. These two provinces were home to two critical components of the network, the central administrative unit and the coordination of Patient-Partner Circles.

One of the distinguishing features of SPOR networks is the inclusion of patient partners in research activities. The study identified several relationships where a patient partner discussed scientific research with a researcher. The study found that several inter-individual discussions about network governance involved a patient partner. The institutionalization of patient partner involvement in large research networks is an evolving practice for which optimal engagement methods are still being explored. As highlighted in a systematic review, there is a need for research on the tangible benefits of patient engagement for researchers and research funders [14]. For example, our findings invite reflection on the opportunity costs of increasing the number and frequency of relationships between researchers and patient partners in research programs. The impact of patient engagement in research on health outcomes and quality of life would also benefit from further study.

We could not identify studies investigating discussion topics within a large research network where patient involvement was incorporated into the structure of the organization. A study similar to ours, which used network analysis methods to study the structure of a transitional research network, examined an Australian Translational Cancer Research Network (TCRN) [21]. However, our respective teams’ objectives were different. Therefore, our findings are not entirely comparable. At first glance, the SPOR DAC network appears to be denser (16% vs. 3.9%) and contains more links (2763 vs. 1658). However, this comparison must be interpreted with caution as the survey question used by the Australian researchers to measure relationships within their network focused on collaboration between members in an activity, project, or event. In contrast, ours measured the frequency of relationships in the network-related activities. Therefore, these different findings may be partly explained by the different measurement approaches or other unknown reasons. There are, however, two parameters on which the two networks can be compared, namely the size of the network and the proportion of members with at least one contact with a network member. After 4 years of life, the DAC SPOR network was extensive (185 members) but smaller than the Australian network (244 members). On the other hand, 18% of the members of the Australian network had no connection with any network member, while all members of the DAC SPOR network were in contact with at least one network member. One possible explanation could be a difference in approach to managing network membership.

The results from these surveys have been presented to the membership and governance committee of the SPOR DAC network and has provided guidance regarding the opportunity to expand engagement with patient partners and to examine how membership from all provinces and territories across Canada can be achieved to provide a broader representation of patient partners and researchers across the country. This SNA is a component of a broader Network Evaluation which has been provided as a component of the SPOR DAC network’s annual reporting to CIHR.

The study’s main limitations are the self-reported nature of the data and the 51.9% participation rate in the 2020 survey, which did not allow for the full examination of the range of relationships within the research network. Considering that several reminders were made and that participation was encouraged by the network’s governance and administration, the low participation rate may indicate a difference in the priority that some members gave to the network’s activities, knowing that several members were also involved in other research programs. The limitation regarding the self-reported nature of the data was mitigated by adding members’ photos in the questionnaire to limit the recall bias that would have occurred for respondents who have difficulty remembering names.

## Conclusions

Funding health research networks is a way for governments to foster the translation of research into concrete solutions for patients and their families and the health system. This social network analysis of one of the Canadian SPOR networks demonstrates how patient partners are engaged in the activities of the research network and to what extent this type of research network involving a plurality of actors with varying interests has a complex relational structure. The study offers an alternative approach to examining network relationships and connectivity when evaluating a research network and hopefully can provide some insight into the identification of trends in the relational structure of such research networks.

## Supplementary Information

**Additional file 1.**

## Data Availability

The datasets generated and analyzed during the current study are publicly available by following this link: https://www.openicpsr.org/openicpsr/project/173801/version/V1/view
