# Supplementary material for: Inter-individual relationships within a Canadian SPOR research network: a social network study
Source: BMC Health Serv Res. 2022 Jul 27;22:955. doi: 10.1186/s12913-022-08343-1 (PMC9326433; doi:10.1186/s12913-022-08343-1)
Supplement: Supplementary file 1 — Additional file 1. [file 12913_2022_8343_MOESM1_ESM.docx]

**Survey questions**

Please indicate **how often you interacted with each of the following individuals who are (or were) formally involved in Diabetes Action Canada**.  

An interaction is defined as **any recalled direct conversation between two individuals**, of any length, through any means of communication (e.g., face-to-face, telephone, email, chatting, texting), which occurred **within the context of Diabetes Action Canada-related activities between April 1, 2019, and March 31, 2020**, inclusively. Please note that there is no interaction between you and Actor *i* if Actor *i* and yourself participated in a joint meeting without talking to each other.

**For each actor, please indicate whether or not you have interacted with him or her between April 1, 2019, and March 31, 2020.**

- If the listed actor is you, please click on "It's me"; 
- If there had been no interaction between you and the listed individual, please click on "No contact"; 
- If you had an interaction with the listed individual, please select on the scale the category that you think best describes the interaction frequency with this individual over the past 12 months (i.e., April 1, 2019, to March 31, 2020). 
 
The **frequency scale** includes five categories: 1. **Daily**, multiple times a day, most days in the last 12 months; 2. **Weekly**, multiple times a week, most weeks in the last 12 months; 3. **Monthly**, from time to time, most months in the last 12 months; 4. **Quarterly**, a few times during the last 12 months; 5. **Yearly**, only once during the last 12 months.

Between April 1, 2019, and March 31, 2020, how often did you interact with **[full name]**(Group: Knowledge Translation) in relation to Diabetes Action Canada?

*[Note: The person's picture appeared on the screen. It is not reproduced here.]*

- It's me
- No contact
- Yearly (only once during the last 12 months)
- Quarterly (a few times during the last 12 months)
- Monthly (from time to time, most months in the last 12 months)
- Weekly (multiple times a week, most weeks in the last 12 months)
- Daily (multiple times a day, most days in the last 12 months)

Q661 Prior to joining the Diabetes Action Canada CIHR SPOR Network, did you previously collaborate with [**full name]** on a research project (e.g., clinical trials, funded projects, quality improvement initiatives)?

*[Note: this question was displayed for identified contacts only].*

- Yes
- No

[Note: the same two questions were asked for each member of the network.]

**Types of interactions**

Now, please **specify the nature of your interactions within Diabetes Action Canada**.

1. **"Scientific Research"** refers to the research process (e.g., grant application, protocol writing, data collection, data analysis) or traditional ways to disseminate research findings (e.g., publications, conferences);
2. **"Training and Mentoring"** refers to student supervision, training or any other educational activity;
3. **"Patient Engagement"** refers to patient partners' participation within the Diabetes Action Canada network (e.g., participation in research committees, collaboration in the research process, management and/or recruitment of patient partners);
4. **"Management and Operations"** refers to the management of projects or activities' implementation (i.e., recruitment, reporting, coordination of services and resources);
5. **"Governance and Coordination"** refers to the strategic decision making for the whole network (e.g., defining the overall mission and objectives of Diabetes Action Canada, program evaluation and planning) or for specific groups (e.g., creating a new group, monitoring projects, coordinating researchers);
6. **"Commercialization of Research Findings"** refers to the commercialization of research products (e.g., licensing, consultancy, intellectual property protection and/or technology transfer processes);
7. **"Transfer of Research Findings"** refers to the dissemination of research results to external actors, excluding commercialization of research and scientific publications (e.g., guidelines, policy briefs, media articles, presentations to decision makers etc.).

**Please, tick all boxes that apply.**

Between April 1, 2019, and March 31, 2020, what did you discuss with **[full name]** in relation to Diabetes Action Canada?

*[Note: the question was asked for each contact identified in the previous question.]*

- Scientific Research
- Training and Mentoring
- Patient Engagement
- Management and Operation
- Governance and Coordination
- Commercialization of Research Findings
- Transfer of Research Findings
- Other
